# Immobilization and detection of platelet-derived extracellular vesicles on functionalized silicon substrate: cytometric and spectrometric approach

**DOI:** 10.1007/s00216-016-0036-5

**Published:** 2016-11-07

**Authors:** Katarzyna Gajos, Agnieszka Kamińska, Kamil Awsiuk, Adrianna Bajor, Krzysztof Gruszczyński, Anna Pawlak, Andrzej Żądło, Artur Kowalik, Andrzej Budkowski, Ewa Stępień

**Affiliations:** 1 Department of Advanced Materials Engineering, M. Smoluchowski Institute of Physics, Jagiellonian University, 11 Łojasiewicza Street, 30-348 Krakow, Poland; 2 Department of Medical Physics, M. Smoluchowski Institute of Physics, Jagiellonian University, ul. S. Łojasiewicza 11, 30-348 Krakow, Poland; 3 Department of Molecular Diagnostics, Holycross Cancer Center, 3 Stefana Artwińskiego Street, 25-734 Kielce, Poland; 4 Department of Biophysics, Faculty of Biochemistry, Biophysics and Biotechnology, Jagiellonian University, 7 Gronostajowa Street, 30-387 Krakow, Poland

**Keywords:** Time-of-flight secondary ion mass spectrometry, Flow cytometry, Biomarkers, Extracellular vesicles, Nanoparticles/nanotechnology

## Abstract

Among the various biomarkers that are used to diagnose or monitor disease, extracellular vesicles (EVs) represent one of the most promising targets in the development of new therapeutic strategies and the application of new diagnostic methods. The detection of circulating platelet-derived microvesicles (PMVs) is a considerable challenge for laboratory diagnostics, especially in the preliminary phase of a disease. In this study, we present a multistep approach to immobilizing and detecting PMVs in biological samples (microvesicles generated from activated platelets and human platelet-poor plasma) on functionalized silicon substrate. We describe the application of time-of-flight secondary ion mass spectrometry (TOF-SIMS) and spectroscopic ellipsometry methods to the detection of immobilized PMVs in the context of a novel imaging flow cytometry (ISX) technique and atomic force microscopy (AFM). This novel approach allowed us to confirm the presence of the abundant microvesicle phospholipids phosphatidylserine (PS) and phosphatidylethanolamine (PE) on a surface with immobilized PMVs. Phosphatidylcholine groups (C_5_H_12_N^+^; C_5_H_15_PNO_4_^+^) were also detected. Moreover, we were able to show that ellipsometry permitted the immobilization of PMVs on a functionalized surface to be evaluated. The sensitivity of the ISX technique depends on the size and refractive index of the analyzed microvesicles.

**Electronic supplementary material:**

The online version of this article (doi:10.1007/s00216-016-0036-5) contains supplementary material, which is available to authorized users.

**Graphical abstract:**
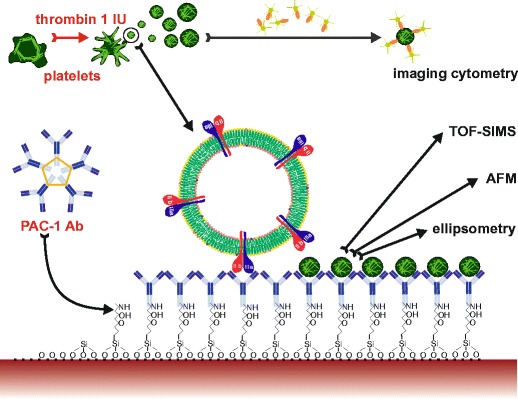
Human *platelets* activated with *thrombin* (in concentration 1IU/mL) generate population of PMVs (platelet derived microvesicles), which can be detected and enumerated with fluorescent-label method (*imaging cytometry*). Alternatively, PMVs can be immobilized on the modified silicon substrate which is functionalized with a specific IgM murine monoclonal antibody against human glycoprotein *IIb/IIIa* complex (*PAC-1*). Immobilized PMVs can be subjected to label-free analyses by means *ellipsometry*, atomic force microscopy (*AFM*) and time-of-flight secondary ion mass spectrometry (*TOF-SIMS*).

## Introduction

Among the various biomarkers that are used to diagnose and monitor cardiovascular disease and its complications, extracellular vesicles (EVs) appear to be one of the most promising targets in the development of new therapeutic strategies and the application of new diagnostic methods [1–3]. Elevated levels of EVs (also called microvesicles or microparticles) have been detected in patients with unstable angina, myocardial infarction, or vascular complications of diabetes mellitus [2, 4, 5]. Most circulating blood EVs originate from platelets, and are therefore usually termed platelet-derived microvesicles (PMVs). They are produced during platelet activation—a physiological process that leads to hemostasis and blood coagulation. As first demonstrated almost two decades ago by Heijnen *et al*., activated platelets release two classes of membrane vesicles: the dominant larger type (100–500 nm in size), known as microvesicles, emerge from the platelet surface in a shedding process, whereas a much smaller type (< 100 nm), named exosomes, are produced during exocytosis [6]. PMVs are postulated to play an important role in thrombus formation and in strengthening clot structures [7].

Detecting circulating PMVs during the preliminary phase of a disease is a considerable challenge for laboratory diagnostics. Despite the availability and accessibility of flow cytometry methods in routine hematology laboratories, most clinical cytometers still have resolutions of around 500 nm. The use of high-sensitivity flow cytometry permits greater analytical resolution, allowing EVs to be resolved down to 400 nm in diameter using a forward scatter detector [8, 9]. The other strategy is to apply the novel method of imaging flow cytometry; for example, to use the ImageStream^X^ Mark II imaging cytometer (ISX), which provides increased sensitivity for smaller EVs. Visual examination of every event that passes through the flow cell allows objects smaller than the optical resolution cut-off of 200 nm to be resolved [10, 11].

Nevertheless, novel label-free methods to detect and quantify PMVs are now available. The use of electrochemical potential-modulated electrochemical impedance spectroscopy (EIS) [12] and other methods that utilize the Coulter principle to determine the absolute size distribution of vesicles in suspension, such as resistive pulse sensing (RPS) [13, 14] and surface plasmon resonance spectroscopy [15], have recently been proposed. Another innovative label-free optical method is grating coupled interferometry (GCI). This sensing system combines the cost-effectiveness, simplicity, and reliability of grating coupled planar optical waveguides with the excellent resolution of interferometric measurements [16]. However, the complexity of body fluids (e.g., whole blood or plasma) still limits such methods to specific applications based on immunodetection techniques.

The main approach used in routine diagnostics is to avoid interference from abundant proteins such as fibrinogen, albumins, immunoglobulins, and trypsin inhibitors. In this approach, immobilizing EVs, especially those generated by platelets, improves the accuracy/fidelity of biosensor-based methods of detecting PMVs [17].

The motivation for the present study was to develop a one-step procedure for detecting EVs that was based on the lab-on-a-chip approach. The first goal was to generate a functionalized surface onto which PMVs are immobilized to facilitate their detection. The second goal was to visualize these immobilized PMVs as appreciable objects and detect them using time-of-flight secondary ion mass spectrometry (TOF-SIMS) and spectroscopic ellipsometry.

In order to achieve these goals, the conventional method of modifying silicon substrates with 3-glycidoxypropyl(trimethoxysilane) (GOPS) and functionalizing with PAC-1 antibody was applied. The PAC-1 antibody recognizes an epitope on the platelet-specific glycoprotein IIb/IIIa (gpIIb/IIIa, αIIbβ3) complex which is exposed on activated platelets and carried by PMVs [4, 18]. This antibody was previously used to functionalize a graphene-oxide-based electrochemical biosensor for detecting PMVs [17]. In our work, TOF-SIMS analysis was applied to demonstrate the complementary analysis of EV lipid composition. This combined approach is extended in the present work, as we adopted the novel technique of imaging flow cytometry (ISX, using an ImageStream^X^ Mark II imaging cytometer) as well as atomic force microscopy (AFM) for EV enumeration and characterization [10, 14]. The advantage of ISX is that it facilitates the visual examination of every single object that passes through the flow cell. Additionally, ISX can detect fluorescent signals from microparticle populations, even when they are smaller than the optical resolution cut-off of 200 nm [11].

The novelty and significance of this approach is that it is a one-step procedure for detecting and profiling lipids in immobilized EVs derived from both human plasma and activated platelets (PMVs). Our work is also original in that it utilizes imaging flow cytometry and AFM for PMV imaging.

## Materials and methods

### Materials and chemicals

Silicon wafers with native oxidized silicon layers (SiOx) were from Si-Mat (Kaufering, Germany). Chemicals used were EGTA and analytical-grade chloroform (POCH, Gliwice, Poland); bovine serum albumin (BSA; cat. no. A2153), glucose, MES, MgCl_2_, NaCl, 3-glycidoxypropyl(trimethoxysilane) (GOPS; cat. no. 440167), PBS (cat. no. 4417), Triton X-100 (cat. no. 9002-93-1), and FITC (fluorescein isothiocyanate)-labeled phalloidin (cat. no. P5282) (Sigma–Aldrich, Munich, Germany); bovine thrombin (Bio Trombina 400, Biomed-Lublin, Lublin, Poland); ethanol, formaldehyde, and toluene (Chempur, Karlsruhe, Germany); phospholipids (1-palmitoyl-2-oleoyl-*sn*-glycero-3-phosphocholine (POPC) and 1-palmitoyl-2-oleoyl-*sn*-glycero-3-phospho-L-serine sodium salt (POPS), cat. nos. 850457P and 840039P, respectively; Avanti Polar Lipids, Inc., Alabaster, AL, USA); flow cytometry antibodies and reagents (annexin V Pacific Blue^TM^ conjugate (cat. no. A35122, Molecular Probes Life Sciences, Paisley, UK), Alexa Fluor^®^ 647 anti-human CD61 antibody and phycoerythrin (PE) conjugated anti-human CD9 antibody (cat. nos. 336408 and 312106, respectively; BioLegend Inc., San Diego, CA, USA), and PAC-1 (cat. no. 340535, BD-Bioscience, Erembodegem, Belgium)); and flow cytometry standardization kits (Sphero^TM^ Flow Cytometry Nano Fluorescent Size Standard Kit (cat. nos. NFPPS-52-4K, NFPPS-0152-5, Spherotech Inc., Lake Forest, IL, USA)).

### Sample collection and PMV preparation

Blood samples for platelet-rich plasma (PRP) preparation were collected in duplicate according to a standardized protocol using the S-Monovette individual blood collection system (Sarstedt AG & Co., Nümbrecht, Germany) from healthy donors (*n* = 3) in the morning (8–11 am) after overnight fasting. The second tube, containing citrate 0.106 M anticoagulant (8.2 mL, cat. no. 01.1606.001), was used for PRP preparation. After phlebotomy, samples were subjected to centrifugation by a Z300K centrifuge (Hermle Labortechnik, Wehingen, Germany) at 165×*g* for 10 min to acquire PRP. After that, platelets were pelleted at 750×*g* for 10 min and twice washed with JNL buffer without Ca^2+^ (130 mM NaCl, 10 mM sodium citrate, 9 mM NaHCO_3_, 6 mM D-glucose, 0.9 mM MgCl_2_, 0.81 mM KH_2_PO_4_, and 10 mM Tris, pH 7.4). To obtain thrombin-generated PMVs, the washed platelets were resuspended in 0.5 ml of JNL buffer with 1.8 mM CaCl_2_ and 1 IU of bovine thrombin (Biomed-Lublin) and incubated for 30 min at 37 °C. After that, the activated platelets were pelleted and the supernatant containing PMVs was subjected to further investigations. Additionally, platelet-poor plasma (PPP) was collected according to protocol A, in which the first centrifugation was performed at 2500× *g* for 15 min at room temperature with a light brake, before the separated plasma was transferred to a new centrifugation tube and centrifuged again as above [19].

#### Fluorescent microscopy observations of platelets

Washed platelets were incubated on prepared siliconized (A) and collagen-coated (B, C) microscopy cover slips in a humidified chamber (30 min at 37 °C). After that, unattached platelets were rinsed three times with a cytoskeleton buffer (CB; 10 mM MES, 150 mM NaCl, 5 mM EGTA, 5 mM MgCl_2_, 5 mM glucose; pH 6.1), fixed with 3.7 % formaldehyde in a phosphate-buffered saline solution (PBS), and finally permeabilized with 0.1 % Triton X-100 in PBS for 30 min. The F-actin cytoskeleton was visualized using FITC-labeled phalloidin. Each coverslip was incubated with 15 μl of this phalloidin-FITC in CB (500 ng/ml) for 15 min at 37 °C in a humidified chamber. After three washes with CB, the stained platelets were observed using an Axiovert 200 fluorescent microscope (Carl Zeiss, Jena, Germany) at 630× magnification.

#### PMV enumeration and visualization

PMVs were counted and characterized using an imaging flow cytometry (ISX) system (ImageStream^X^ Mark II, Amnis Corporation, Seattle, WA, USA) equipped with four lasers (wavelengths: 405, 488, 642, and 785 nm), and the resulting data were analyzed with the IDEAS 6.0 software package (Amnis Corporation). During calibration and in experiments with biological material, a maximum power of 200 mW was used for the blue laser (488 nm), 150 mW for the red laser (642 nm), 120 mW for the violet laser (405 nm), and 70 mW for the 785 nm laser (SSC). Pictures were collected at the highest optical magnification (60×) with a numerical aperture of 0.9 and an image resolution of approximately 0.3 × 0.3 microns/pixel [10]. Since the ISX system was equipped with two CCD sensors (each with six channels of signal detection), two channels of transmitted light (bright field, BF) were required to achieve spatial coordination between the matrices. The intensity of the background for the BF channel was set to 800 for both matrices. Acquisition was performed for 5,000 objects. Calibration for MVs was performed using Sphero^TM^ Flow Cytometry Nano Fluorescent Size Standard Kits, including five categories of microbeads (0.13, 0.22, 0.45, 0.88, and 1.33 μm in diameter) labeled with FITC to avoid interference when linking with the acquired files. Five microliters of PMV suspension diluted up to 150 μl volume in PBS and annexin V binding buffer. Then, 5 μl of each antibody and annexin V were added and the mixture was incubated at room temperature for 15 min. The acquired files were virtually merged with the previous calibrator data files and analyzed with the IDEAS software package.

### Preparation of multilamellar and unilamellar liposomes

Multilamellar and/or unilamellar liposomes consisting of POPC (1 mM) alone or POPC (0.95 mM) and POPS (0.05 mM) were prepared according to previously described methods [20–22]. Briefly, a mixture of the selected lipids at desired concentration was dissolved in chloroform saturated with argon to prevent oxidation. Chloroform was then evaporated with a stream of argon/nitrogen gas, and the lipid film that formed on the bottom of the test tube was thoroughly dried under reduced pressure for 12 h. A buffer solution was added to the dried lipids at a temperature higher than the main phase-transition temperature of the lipids used (room temperature was appropriate for the lipids used in this work), and the mixture was vortexed vigorously. To obtain unilamellar liposomes, the suspension was extruded using an extruder with needles (cat. no. 610000, Avanti Polar Lipids, Inc.) and filtered using filters with pore diameters of 0.1 μm and 0.2 μm (cat. nos. 610005 and 61006, Whatman, GE Healthcare Ltd., Little Chalfont, UK) above the phase-transition temperature of the lipids. The size distribution and stability of the liposomes were analyzed in 10× dilution using a Zetasizer Nano S particle size analyzer (Malvern Instruments, Malvern, UK).

### Surface functionalization to facilitate the immobilization of PMVs

Next, a multistep method was carried out to functionalize the silicon surface with PAC-1 antibodies, thus promoting the immobilization of PMVs. In the first step, Si substrates were washed with ethanol and toluene and then cleaned and hydrophilized with oxygen plasma. The second step involved silanizing the substrates by immersing them in 1 % (v/v) GOPS in anhydrous toluene for 1 h, washing the substrates in a sequence of toluene and ethanol baths, and finally drying them under a stream of N_2_. Modification of the silicon surface with epoxysilane facilitated direct covalent bonding to proteins (antibodies) [23]. In the third step, PAC-1 antibodies were immobilized on the silanized silicon substrates by incubating them in a 100 μg/mL solution of PAC-1 in PBS (pH 7.4) for 24 h. After the incubation and washing steps, blocking was performed in the fourth step by incubating the substrates in BSA (10 mg/mL) for 1 h. Finally, the functionalized surfaces were incubated for 1 h with human plasma or PMVs from activated platelets. As a negative control, substrate samples were incubated with 100 μg/mL BSA for 24 h instead of incubating with PAC-1 (stage 3), and then stages 4 and 5 were performed. All substrates were washed with PBS buffer and distilled water and dried under a stream of N_2_ before surface characterization.

### Surface characterization

#### Atomic force microscopy

Topographic micrographs of surfaces silizanized with GOPS, functionalized with PAC-1 antibodies, blocked, and with PMVs immobilized on them were recorded in air using an Agilent (Santa Clara, CA, USA) 5500 microscope working in a noncontact mode. AFM probes with a spring constant of 2 N/m, a tip radius of <7 nm, and a resonant frequency of about 70 kHz were used. For all samples, the mean height distribution in the AFM image and the doubled width at half-maximum of a radially averaged autocorrelation function were taken as the AFM height and feature size, respectively [24, 25]. In turn, for the surface exposed to PMVs, the AFM height and feature size were estimated from cross-sections through *n* = 25 individual microvesicles visualized on AFM images. AFM images were analyzed with the WSxM software provided by Nanotec Electronica S.L. [26] (downloadable at http://www.nanotec.es).

#### Spectroscopic ellipsometry

The effective ellipsometric thickness of the molecular overlayer on each silicon substrate after the successive modification and immobilization steps was evaluated with a Sentech (Berlin, Germany) SE800 spectroscopic ellipsometer. Spectra of two ellipsometric angles (*Ψ* and Δ) relating to the amplitude of and phase difference between the parallel and perpendicular components of the polarized light beam following reflection from a surface were recorded. The measurements were performed over the wavelength range 320–700 nm and at a fixed angle of incidence of 70°. Results were analyzed with the SpectraRay 3 software. The effective thickness of the molecular layer was estimated by fitting a model assuming uniform layers to each pair of ellipsometric angles. This uniform-layers model used the Cauchy dispersion model, which describes the refractive index (*n*) as a function of the wavelength (*λ*): *n* = *A*+ *B*/*λ* ^2^ + *C*/*λ* ^4^. A three-layer model (silicon substrate/mixed SiOx and GOPS layer/protein layer/PMVs overlayer) was applied. Fixed refractive index values of *n* = 3.87 for Si, *n* = 1.46 for both SiOx and GOPS [27, 28], *n* = 1.53 for biomolecules (proteins and lipids) [29–31], and *n* = 1.39 for PMVs [8, 32] were used. A mixed SiOx and GOPS layer of constant thickness 2.88 ± 0.14 nm (value obtained from fitting measurements performed on a bare GOPS-modified silicon substrate) was assumed in order to fit the thickness of the biomolecular layer.

#### Time-of-flight secondary ion mass spectrometry

After functionalization and PMV immobilization, surface molecular composition analysis of the silicon substrates was performed using a TOF.SIMS 5 instrument (ION-TOF GmbH, Münster, Germany) equipped with a 30-keV bismuth liquid metal ion gun. Bi_3_^+^ clusters were used as primary ions, applying an ion dose density of <10^12^ ion/cm^2^ to ensure static mode conditions. A low-energy electron flood gun was used for charge compensation. Positive high-resolution mass spectra (with minimal mass resolution (*m*/Δ*m*) > 6000 at C_4_H_5_^+^) were acquired from several nonoverlapping 100 μm × 100 μm regions (with a resolution of 128 × 128 points). The TOF-SIMS data were normalized with respect to the total ion intensity.

## Results and discussion

### Visualization of PMVs and liposomes by imaging flow cytometry

**Fig. 1a–g:**
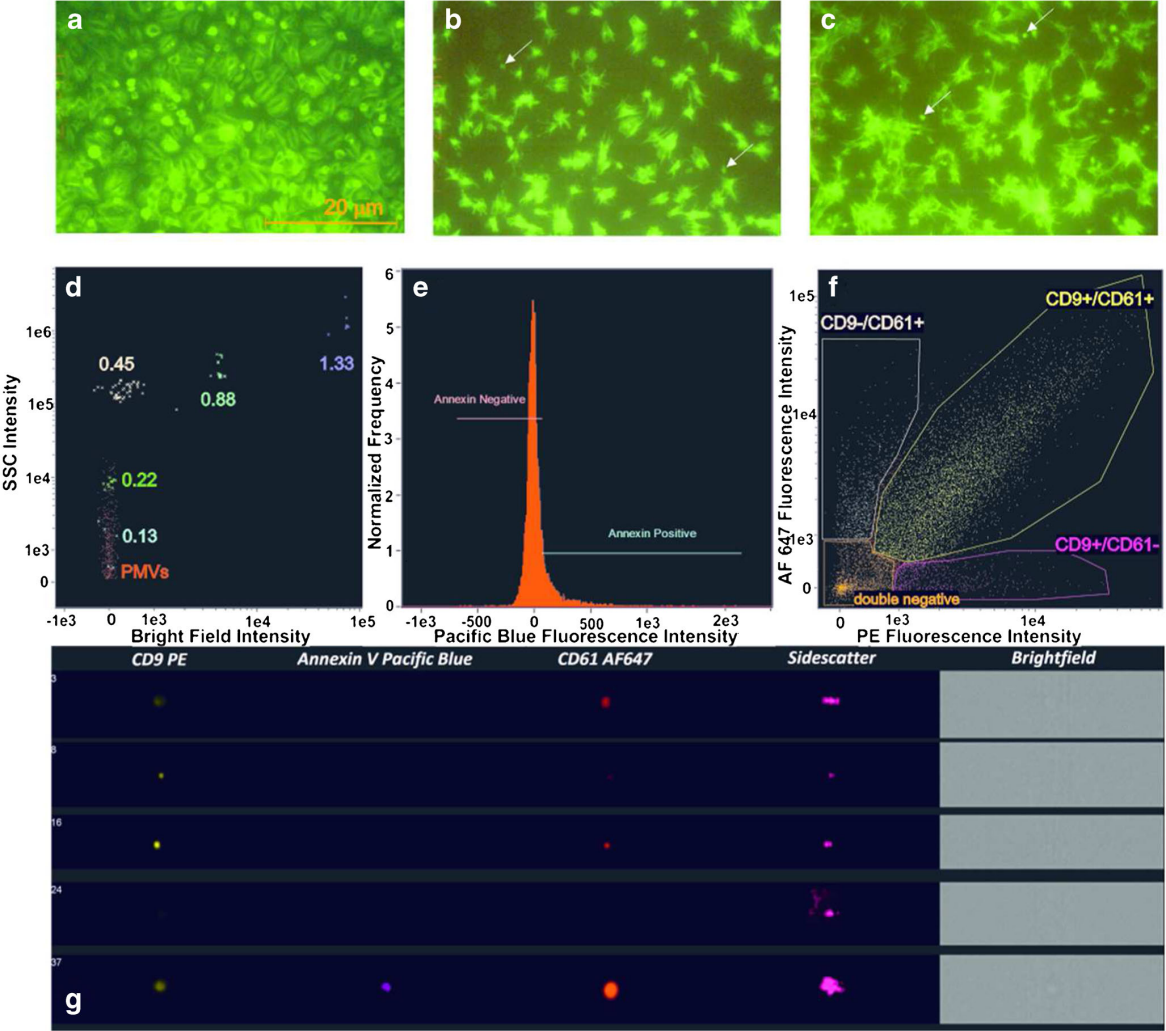
Optical images of platelets and platelet-derived microvesicles (PMVs). Platelets obtained from PRP (platelet-rich plasma) were placed on siliconized (**a**) and collagen-coated (**b**, **c**) glass slides. Phalloidin staining revealed specific actin cytoskeleton organization. Thrombin activation produced more phalloidin-stained spots (**c**).* Arrows* indicate actin streaks from activated platelets, which correspond to actin-rich PMVs. ImageStream^X^ Mark II cytometry showed that most of the PMVs were gated between 0.13 and 0.22 μm in sidescatter (SSC) and bright-field channels (**d**). A minority (∼30 %) of the PMVs were positive for annexin V (**e**); most of the PMVs were double-positive for the antigens CD9 (exosome-specific) and CD61 (platelet-specific) (**f**). Selected images of microvesicles are shown in **g**.* AF* Alexa Fluor,* PE* phycoerythrin

Platelets present in the PRP were visualized via fluorescent microscopy. A phalloidin staining method revealed specific actin cytoskeleton organization. When unactivated platelets were placed on a siliconized surface, the abundant actin filaments traced out a regular distribution resembling cobblestones, comprising central rings with a thin peripheral zone (Fig 1a). A collagen-coated surface caused platelet activation and PMV release (Fig 1b, c). Actin filaments were stellate with actin streaks, which became more distinguishable after thrombin activation (Fig 1c). Moreover, platelet aggregates were observed after thrombin treatment. The average unactivated adhered platelet diameter was between 1 and 3 μm (Fig. 1c), but was less for activated platelets (Fig 1b, c).

The ImageStream^X^ Mark II imaging cytometer detected three EV populations in the supernatent containing activated platelets. The entire number of PMVs in 500 μl of supernatant was 20,612 (± 10,664) counts/μl. Most (98.5 %) of the PMVs were gated between 0.13 and 0.22 μm according to the calibration procedure used in the SSC and bright-field channels (Fig. 1d) [10]. The immunofluorescent staining method demonstrated that a minority of the small PMVs (∼30 %) were positive for annexin V (i.e., they presented phosphatidylserine, PS); nevertheless, most of the microvesicles (∼60 %) were double-positive for the antigens CD9 (exosome-specific) and CD61 (platelet-specific) (Fig. 1e, f). This observation was confirmed by a single microvesicle analysis (Fig. 1g). Such diversity of EV antigens is commonly observed in biological samples and may be useful for EV profiling [10, 15].

**Fig. 2a–d:**
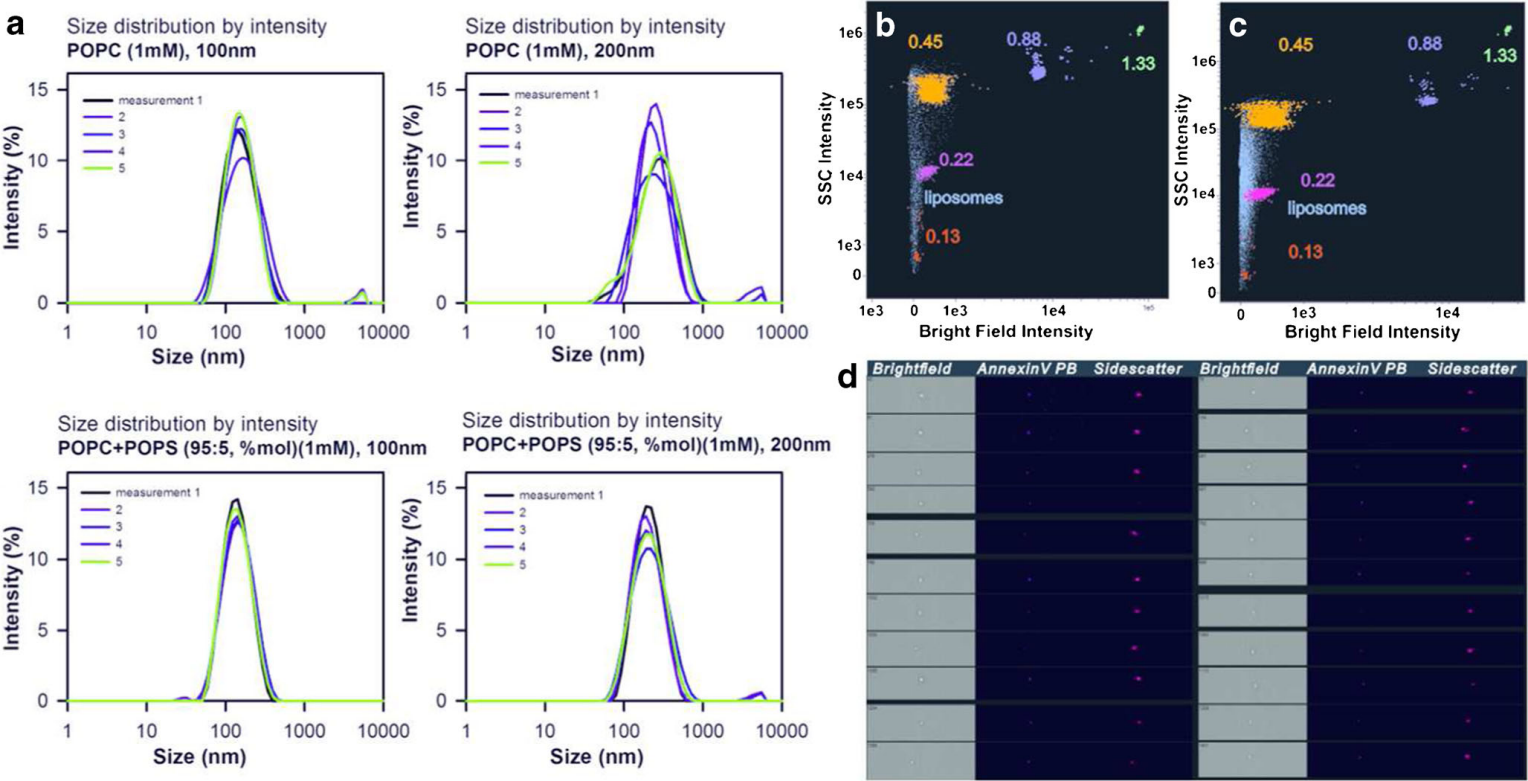
Visualization of multilamellar and/or unilamellar POPC and POPC/POPS liposomes. The size distribution and stability of the liposomes were analyzed using a Malvern Zetasizer Nano S particle size analyzer. The POPC/POPS liposomes were more homogeneous in size and more stable than the pure POPC liposomes (**a**). Overlaying 100-nm (**b**) and 200-nm (**c**) POPC/POPS liposomes (*blue*) with the polystyrene nanobeads (0.13, 0.22, 0.45, 0.88, and 1.33 μm) acquired by ISX demonstrated that ISX resolved the 200-nm liposomes better. The difference in relative scatter intensity between these two types of particles was only distinguishable for the 200-nm liposomes. Representative images of objects stained with annexin V Pacific Blue showed that the annexin V fluorescence was localized on liposomes (**d**).* PB* Pacific Blue,* POPC* 1-palmitoyl-2-oleoyl-*sn*-glycero-3-phosphocholine,* POPS* 1-palmitoyl-2-oleoyl-*sn*-glycero-3-phospho-L-serine sodium salt

In order to evaluate the sensitivity of the ISX system, two populations of POPC and POPC/POPS liposomes were examined: 100-nm and 200-nm liposomes. The pure POPC liposomes were less stable than the POPC/POPS liposomes, and the 200-nm liposomes were more varied in size than the 100-nm liposomes (Fig. 2a). ISX discrimination of liposomes with respect to the SSC/bright-field distribution demonstrated differences in relative scatter intensity between the polystyrene calibrator beads and liposomes (Fig. 2b, c). The refractive index of the PMVs is *n* = 1.39, which is why the site scatter intensity is weaker for lipid vesicles, including liposomes [8, 32]. Moreover, the smaller liposomes (100 nm) were less distinguishable than the larger liposomes, confirming the notion of a physical optical resolution cutoff of 200 nm for flow cytometry methods [11]. In order to look for a correlation between the fluorescence signal and SSC intensity, annexin V staining of the POPC/POPS liposomes was performed. ISX analysis did not find any relationship between those two variables (see Figs. S1 and S2 in the “Electronic supplementary material,” ESM).

### Visualization of immobilized PMVs by AFM

**Fig. 3a–b:**
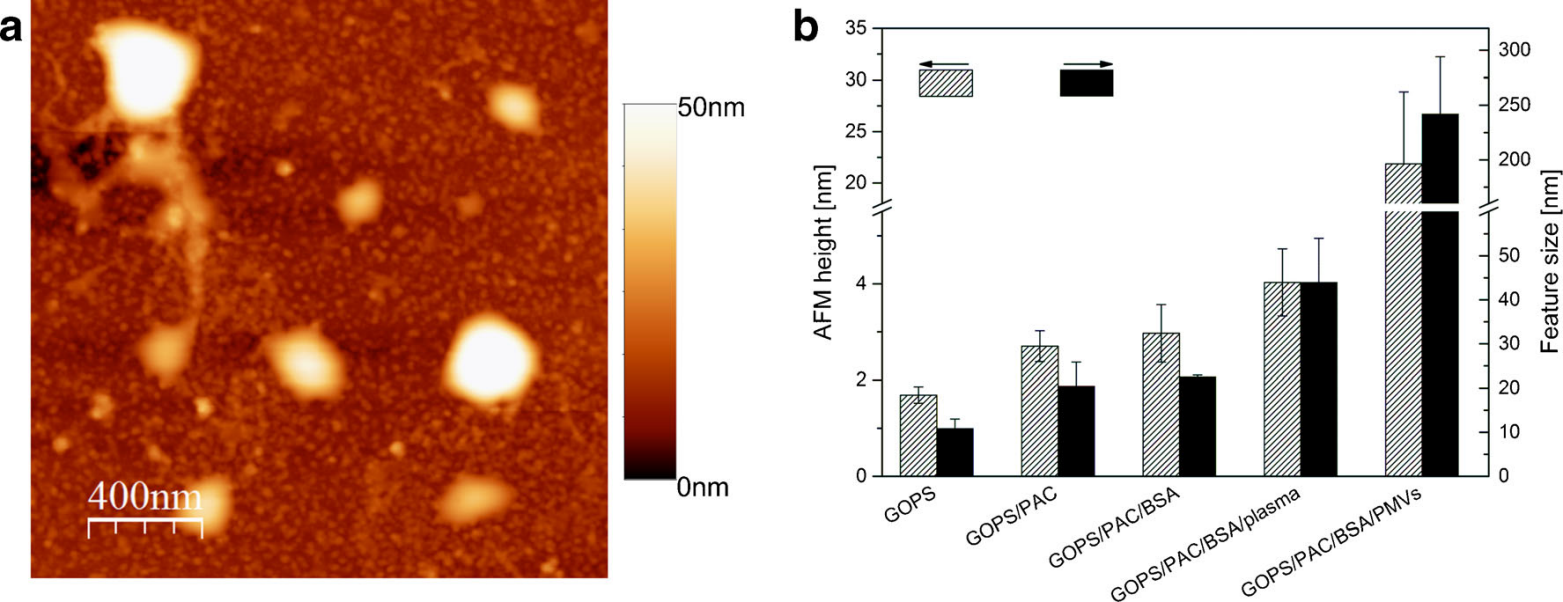
Representative AFM topographic micrograph of PMVs immobilized on a functionalized silicon substrate (**a**); scan size is 2 × 2 μm^2^. Plot (**b**) showing values of some structural parameters—AFM height and feature size—of the biomolecular layer, as determined after surface functionalization and microvesicle immobilization.* Error bars* are standard deviations determined from five AFM images of the same surface and measurements of 25 individual PMVs.* BSA* bovine serum albumin,* GOPS* 3-glycidoxypropyl(trimethoxysilane),* PAC* IgM anti-glycoprotein IIb/IIIa,* PMVs* platelet-derived microvesicles

AFM imaging of silicon substrate functionalized with PAC-1 antibodies showed the successive immobilization of PMVs released from thrombin-activated platelets (Fig. 3a). Smooth oval objects ranging in size from 100 to 250 nm were detected. These findings were consistent with the results of imaging flow cytometry, in which dimensions between the 0.13-μm and 0.22-μm calibration beads were usually observed.

In order to characterize the molecular overlayer formed during functionalization and PMV immobilization, nanostructural parameters such as AFM height and feature size were determined (Fig. 3b). The GOPS-modified silicon substrate was characterized by an AFM height of 1.7 (± 0.2) nm and a feature size of about 11 (± 2) nm, which are in good accord with literature reports of silanized surfaces of this type [23, 24, 33]. Incubation of the GOPS-modified surface with PAC-1 antibody led to surface coverage and a distinct increase in AFM height (of about 1.0 nm). The mean surface feature size was 22 (± 5) nm, which corresponded to the apparent size of the immobilized IgG monolayer (about 25 nm), and was thought to be due to bordering caused by the AFM tip [34, 35], indicating that individual monomers of pentameric IgM were resolved in the AFM image. Subsequent blocking of free sites on the surface with BSA barely changed the surface coverage, since the nanostructural parameter values were largely unchanged after the blocking procedure. Incubation of the PAC-1-functionalized surface with PMVs resulted in microvesicle immobilization. PMVs were clearly recognizable in the AFM image, and the average height and size of the recorded PMVs were 32 (± 7) nm and 230 (± 70) nm, respectively (Fig. 3b). In turn, incubation of the functionalized surface with human plasma led to a distinct change in the molecular overlayer. Increases in both the AFM height (of about 1 nm) and the feature size (to 40 (± 10) nm) were observed, revealing that plasma molecules had been adsorbed. However, individual intact microvesicles were not visible in the recorded AFM images after exposure to plasma. The most abundant protein in human plasma is fibrinogen, the concentration of which ranges between 2 and 3 g/mL. PAC-1 is a specific IgM antibody against the gpIIb/IIIa (α2β3) integrin complex, a receptor for fibrinogen and von Willebrand factor [36]. It is possible that some of the immobilized integrin complexes from plasma were able to bind to fibrinogen on the functionalized surface. Another explanation is the formation of fibrin and its combination with proteins in the plasma to form the molecular overlayer [37].

### Ellipsometry-determined thickness of the biomolecular layer on the functionalized surface

**Fig. 4:**
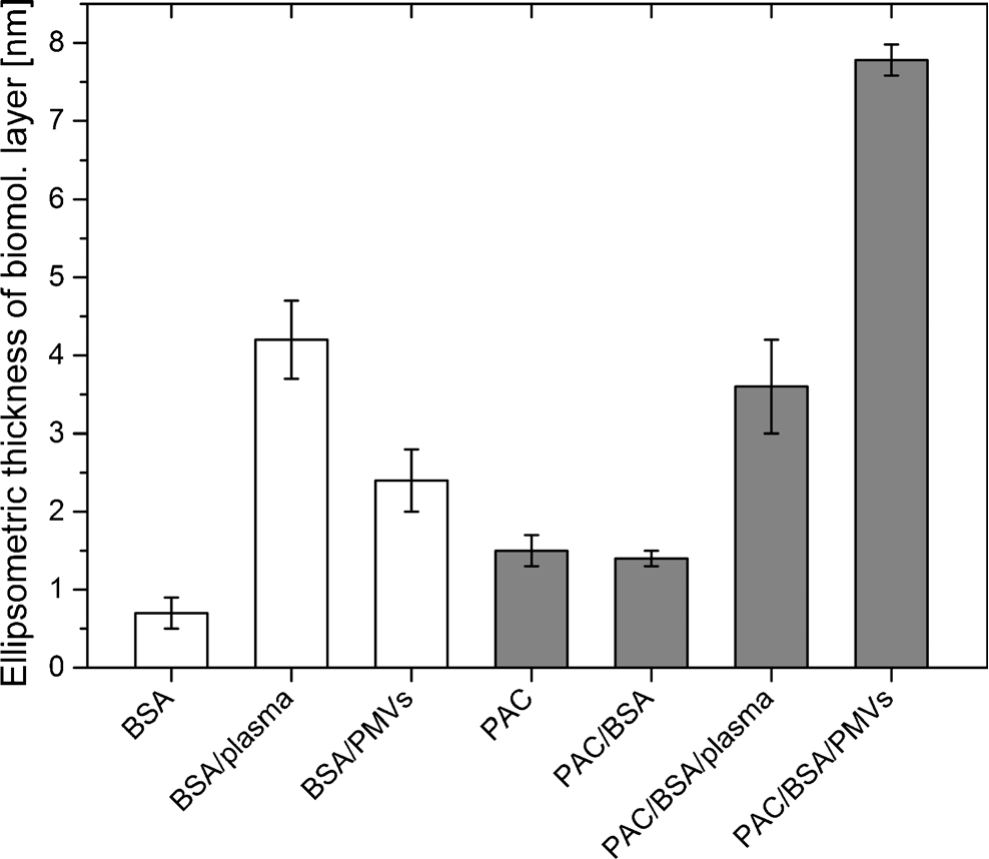
Ellipsometric thickness of the biomolecular layer formed on a silanized silicon substrate following functionalization of the substrate and microvesicle immobilization (*gray columns*), as well as that for the negative control (*white columns*). The evaluated thickness of the GOPS layer was 0.78 ± 0.04 nm.* BSA* bovine serum albumin,* GOPS* 3-glycidoxypropyl(trimethoxysilane),* PAC* IgM anti-glycoprotein IIb/IIIa,* PMV*s platelet-derived microvesicles

The application of spectroscopic ellipsometry to determine the thickness of the molecular overlayer formed on each silicon substrate allowed us to examine the effects of each step in the functionalization of the silicon surface and to detect immobilized microvesicles. Ellipsometry is considered to be a sensitive method if the thin-film thickness is within the wavelength range of the light used for measurements—usually 5–1000 nm. This range is useful and produces good results in EV experiments. Since immobilized PMVs do not form a coherent layer, the effective thickness is related to the amount of PMVs on the surface rather than the height of the immobilized PMV measured with AFM. Firstly, the thickness of the native SiOx layer, 2.1 (± 0.1 nm), was obtained for the cleaned and hydrophilized silicon substrate. Modification with GOPS yielded a monomolecular silane layer with a thickness of 0.76 (± 0.04) nm, which is similar to corresponding values reported in the literature [38]. The subsequent immobilization of PAC-1 antibody led to surface coverage with an ellipsometric thickness of the protein overlayer of 1.2 (± 0.2) nm. This value allowed us to estimate the PAC-1 antibody surface density using the protein mass density (1.37 g/cm^3^) as a scaling factor. The evaluated surface coverage of 1.6 (± 0.3) mg/m^2^ is relatively high compared to reported coverage values for protein immobilized on GOPS-modified surfaces [23, 24] . This confirms the effective immobilization of PAC-1 antibody on the silicon substrate by covalent bonding. Blocking the free surface sites with BSA hardly changed the thickness of the protein overlayer. This agrees with our AFM results, which also reveal that blocking has almost no impact. The incubation of PAC-1 antibody-functionalized substrates with plasma and the isolated PMV solution led to significant increases in the ellipsometric thickness of the biomolecular layer of 2.2 nm and 6.4 nm, respectively. When the isolated PMV solution was used, the estimated surface coverage with PMVs, as evaluated by assuming the constant microvesicle height taken from AFM measurements, was about 10 %, confirming the effective binding of the PMVs with the surface. In order to examine the impact of nonspecific adsorption on the measured ellipsometric thickness, a negative control was performed, which involved incubating a BSA-covered substrate with plasma and then in a solution of isolated PMVs. The results obtained (white columns in Fig. 4) confirm that the PMVs highly specifically bind to the functionalized substrates, but they also reveal high levels of nonspecific plasma molecule adsorption. The nonspecific binding of plasma proteins was gauged by comparing the difference in ellipsometric thickness between PAC/BSA/plasma and PAC/BSA with the difference in ellipsometric thickness between BSA and BSA/plasma. This suggests that the ellipsometric measurement of the thickness of the molecular overlayer on a functionalized silicon substrate is a promising approach for estimating the amount of microvesicles in a solution of isolated PMVs rather than in plasma. The average number of PMVs according to ISX was about 2,000 counts/μL.

### Detection of immobilized PMVs by TOF-SIMS

**Fig. 5:**
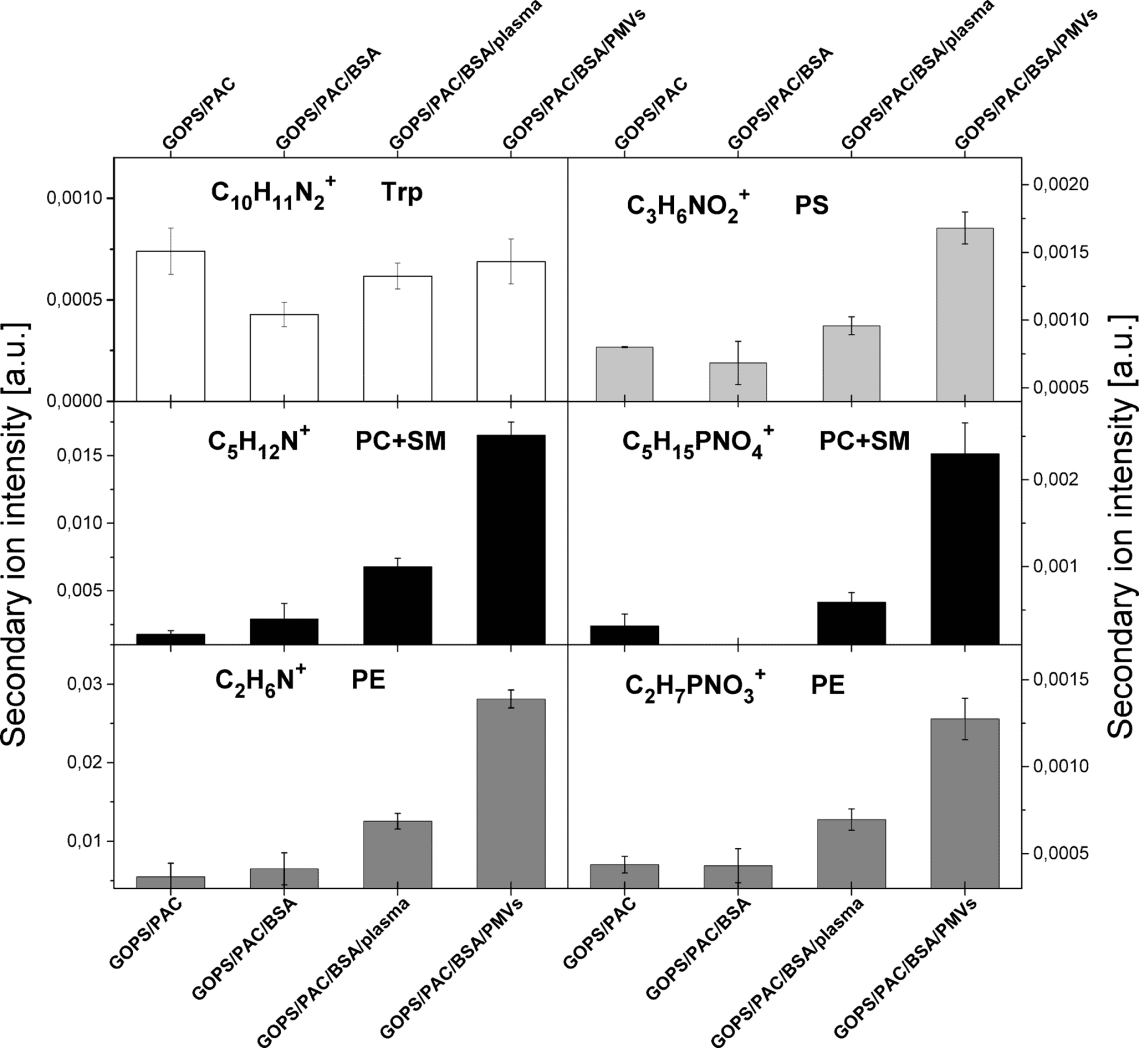
TOF-SIMS analysis of the biomolecular layer that formed on a silanized silicon substrate after functionalization and microvesicle (PMV) and plasma immobilization. Normalized intensities of secondary ions characteristic for amino acids and phospholipids: C_10_H_11_N_2_^+^, tryptophan fragment; C_3_H_6_NO_2_^+^, phosphatidylserine (PS) fragment; C_5_H_12_N^+^, choline fragment; C_5_H_15_PNO_4_^+^, phosphocholine head group; C_2_H_6_N^+^, dimethylamide group; C_2_H_7_PNO_3_^+^, phosphatidylethanolamine (PE) fragment.* BSA* bovine serum albumin,* GOPS* 3-glycidoxypropyl(trimethoxysilane),* PAC* IgM anti-glycoprotein IIb/IIIa,* PC* phosphatidylcholine,* PE* phosphatidylethanolamine,* PMVs* platelet-derived microvesicles,* PS* phosphatidylserine,* SM* sphingomyelin,* Ttp* tryptophan (an amino acid)

TOF-SIMS spectrometry was applied to examine the molecular compositions of the functionalized silicon substrates and to perform the direct chemical detection of immobilized microvesicles. TOF-SIMS ion fragments characteristic of proteins and membrane lipids were analyzed (Fig. 5). The ion fragment C_10_H_11_N_2_^+^ (*m*/*z* 159) of tryptophan [39], an amino acid that is much more abundant in the antibody (2.0 % for IgG [40]) than albumins are (0.2 % for both BSA [41] and HSA [39]), was taken as a TOF-SIMS signal that is characteristic of the antibody. The intensity of this signal remains stable after blocking and microvesicle immobilization, confirming the irreversible functionalization of the surface with the PAC-1 antibody. Signals originate from different membrane phospholipids: C_3_H_6_NO_2_^+^ (*m*/*z* 88) from the amino acid serine, which is present in phosphatidylserine (PS) [42]; C_5_H_12_N^+^ (*m*/*z* 86) and C_5_H_15_PNO_4_^+^ (*m*/*z* 184) from phosphocholine [42, 43], which is present in phosphatidylcholine (PC) and sphingomyelin (SM); and C_2_H_6_N^+^ (*m*/*z* 44) and C_2_H_7_PNO_3_^+^ (*m*/*z* 124), which are characteristic of phosphatidylethanolamine (PE) [42]. The excessive increases in all of these signals after exposure to isolated PMVs confirm the effective immobilization of PMVs on the surface and the presence of phospholipids on the microvesicle membrane. The slight increases observed after exposure of the functionalized silicon surface to the plasma indicate that circulating plasma microvesicles are immobilized and that there is considerable nonspecific adsorption of other plasma molecules. The analysis presented here indicates that TOF-SIMS is a promising technique for detecting PMVs and for characterizing EV lipid composition.

### Limitation of the study

We are aware that our study has some limitations that may influence the interpretation of the findings of our research. One potential limitation is that the entire population of EVs from human plasma and from activated platelets was used. There are a number of studies showing that platelets can release a heterogeneous population of vesicles, including plasma-membrane-derived microvesicles (PMPs), exosomes, and other EV subcategories [44, 45]. EV formation can be stimulated by platelet agonists (mostly thrombin and collagen), mimicking true hemostasis. Among a number of universal platelet activators, Ca^2+^ ionophores also appear to stimulate EV formation in platelets [45]. In our study, we used the commonly accepted platelet agonist thrombin at a concentration of 1 IU. This activator is less effective than a Ca^2+^ inonophore, especially for exosome formation. However, its stimulatory activity is stronger than those of ADP and bacterial lipopolysaccharide [45]. It is also important to note that protein concentration and protein cargo vary depending on microvesicle stimulation and the separation methods used. Gradient centrifugation or single-step isolation by size-exclusion chromatography allows enriched and more homogeneous subpopulations of EVs to be separated [45, 46]. The resulting fractions are free of abundant plasma proteins and are more regular in terms of size or density [45, 46]. Nevertheless, the PMV-specific surface antigen glycoprotein IIb/IIIa has been detected in every platelet EV subpopulation using mass spectrometry [45].

## Conclusions

Activated platelets are the main source of microvesicles in physiological and pathological processes, although the conditions caused by disease are the most important stimuli for EV shedding [2, 6, 18, 47]. Because of this, EVs are considered to be the most promising potential biomarker for various diseases, and the use of EVs as a biomarker has led to some particularly spectacular clinical outcomes in cardiovascular trials [3, 4].

The first goal of the present work was to create an appropriate surface for EV detection. The need for a highly specific sensor for EVs, especially PMVs, is an emerging issue in laboratory diagnostics and biomedical engineering [15, 17]. A variety of methods, including optical (nanoparticle tracking analysis, high-resolution flow cytometry) and non-optical (AFM, transmission electron microscopy, impedance-based measurements, grating coupled interferometry) imaging techniques have recently been developed and tested, but they cannot be performed rapidly in many laboratories or used in routine diagnostics [8–16]. An approach based on an immunosensor surface could permit the efficient and specific capture of EVs for further evaluation [15, 17]. In our study, we developed a method of restraining both PMVs and circulating plasma microvesicles in which monoclonal IgM antibody was immobilized on a GOPS-modified silicon surface. By creating this gpIIb/IIIa integrin complex-specific surface, we were able to immobilize integrin-positive circulating PMVs, which facilitated the characterization of the size (diameter), amount, and lipid composition of the PMVs.

This work is the first to demonstrate phospholipid components of PMVs using a TOF-SIMS approach. We confirmed the presence on the surface with immobilized PMVs of EV-specific phosphatidylserine (PS); phosphatidylethanolamine (PE), which is abundant in the plasma membrane; and sphingomyelin (SM), which is abundant in platelets. While the phospholipidomes of platelets and PMVs were found to be qualitatively and quantitatively similar, the lipid fraction of blood microparticles was observed to be enriched with phosphatidylcholine lipids in another study [48]. That finding contrasts with the results of our TOF-SIMS analysis, in which stronger signals were obtained from PC and PE lipids on a surface with immobilized PMVs. Another interesting finding of our work is the confirmation of the presence of SM in PMVs. High concentrations of SM in the platelet lipid fraction have previously been observed using biochemical methods [49]. Unexpectedly, we did not observe a significant increase in PS and PE on the surface with immobilized human plasma. It was previously demonstrated that 20 % of the native microvesicles in human plasma are derived from other components, such as erythrocytes, leucocytes, and endothelial cells [2]. We believe that circulating plasma microvesicles have different lipid components from those in PMVs [48]. Our findings allow fresh insight into microvesicle lipidomics, and can be applied in further investigations of EVs that aim to characterize MVs with different origins and identify specific biomarkers [3].

Finally, it is worth noting that imaging flow cytometry is a refraction-limited technique. The use of polystyrene beads for cytometer calibration does not overcome this inadequacy, and the application of a more congruent material (calibrated liposomes) highlighted this limitation for smaller objects (<200 nm in size). Because of this limitation of ISX, ellipsometry could be a more useful technique for analyzing EV immobilization.

## Electronic supplementary material

Below is the link to the electronic supplementary material.

Esm 1 (PDF 453 kb)
