# Supplementary material for: Immobilization and detection of platelet-derived extracellular vesicles on functionalized silicon substrate: cytometric and spectrometric approach
Source: Anal Bioanal Chem. 2016 Nov 7;409(4):1109–19. doi: 10.1007/s00216-016-0036-5 (PMC5258792; doi:10.1007/s00216-016-0036-5)
Supplement: Supplementary file 1 — (PDF 453 kb) [file 216_2016_36_MOESM1_ESM.pdf]

Analytical and Bioanalytical Chemistry

Electronic Supplementary Material

Immobilization and detection of platelet-derived extracellular vesicles on functionalized silicon substrate: cytometric and spectrometric approach

Katarzyna Gajos, Agnieszka Kamińska, Kamil Awsiuk, Adrianna Bajor,
Krzysztof Gruszczyński, Anna Pawlak, Andrzej Żądło, Artur Kowalik, Andrzej Budkowski,
Ewa Stępień

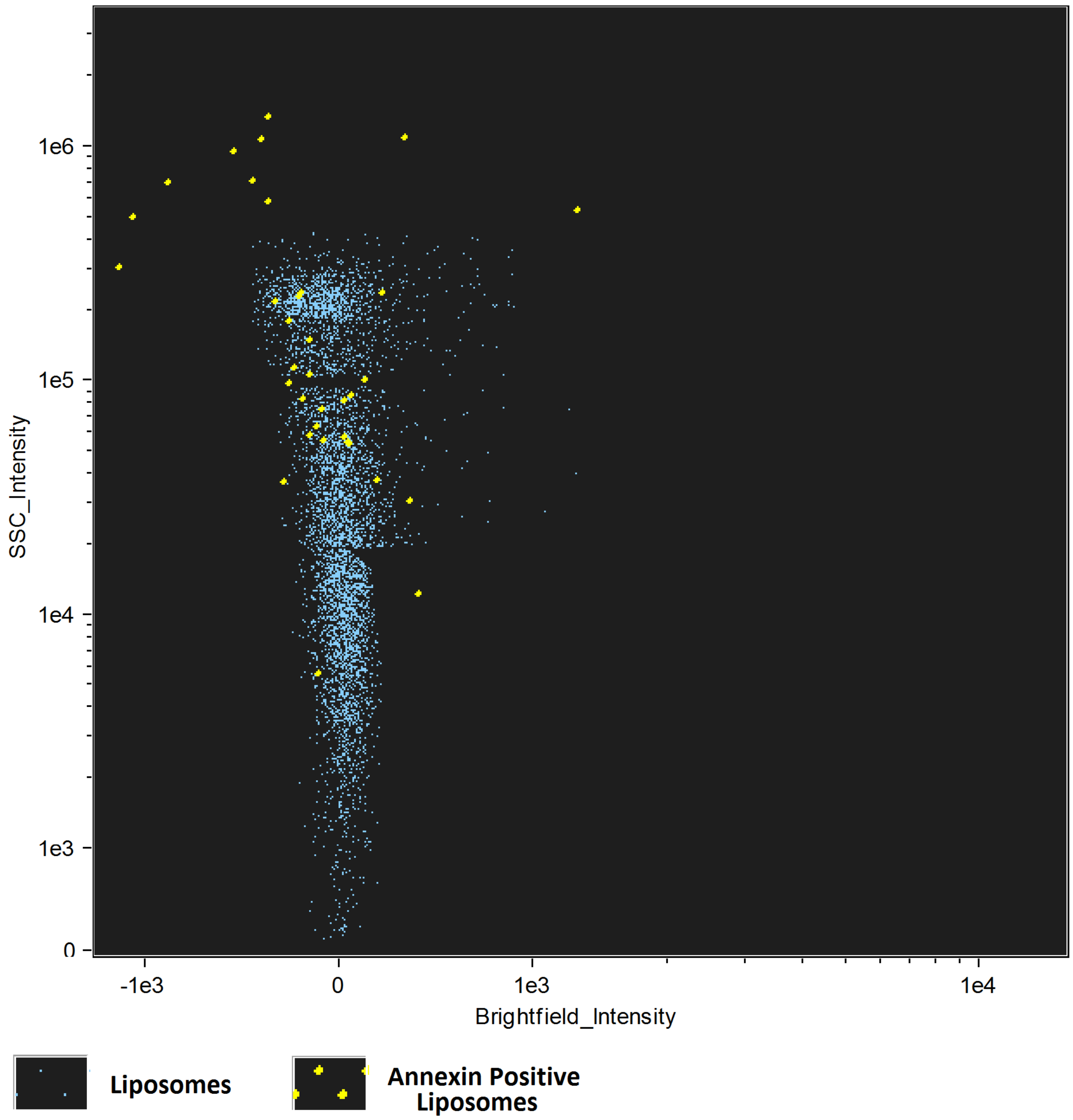

Fig. S1 ImageStreamX Mk II cytometery analysis of Annexin V stained 200 nm liposomes composed with phospholipids: 95% 1-palmitoyl-2-oleoyl-sn-glycero-3-phosphocholine (POPC) and 5% 1-palmitoyl-2-oleoyl-sn-glycero-3-phospho-L-serine sodium salt (POPS)

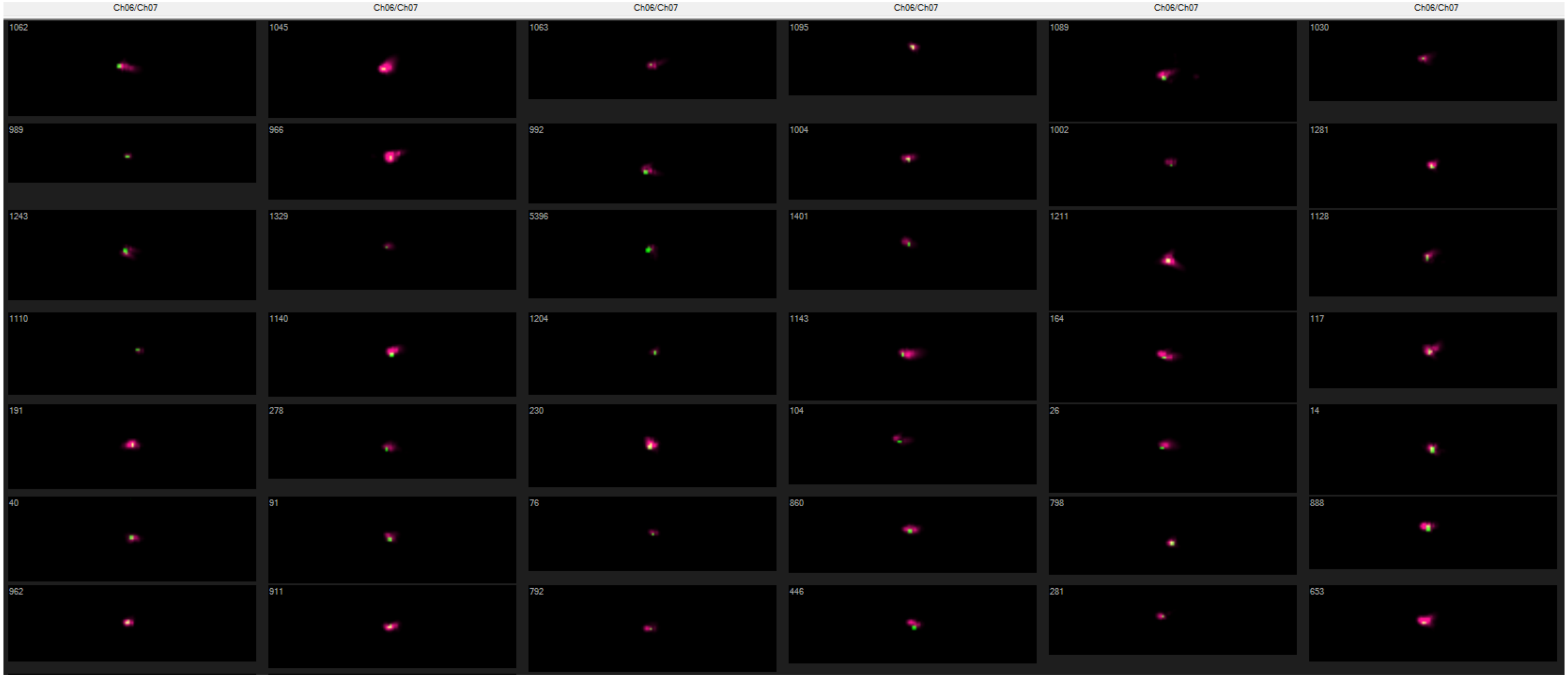

Fig. S2 Gallery of ISX images of 200 nm POPC/POPS liposomes

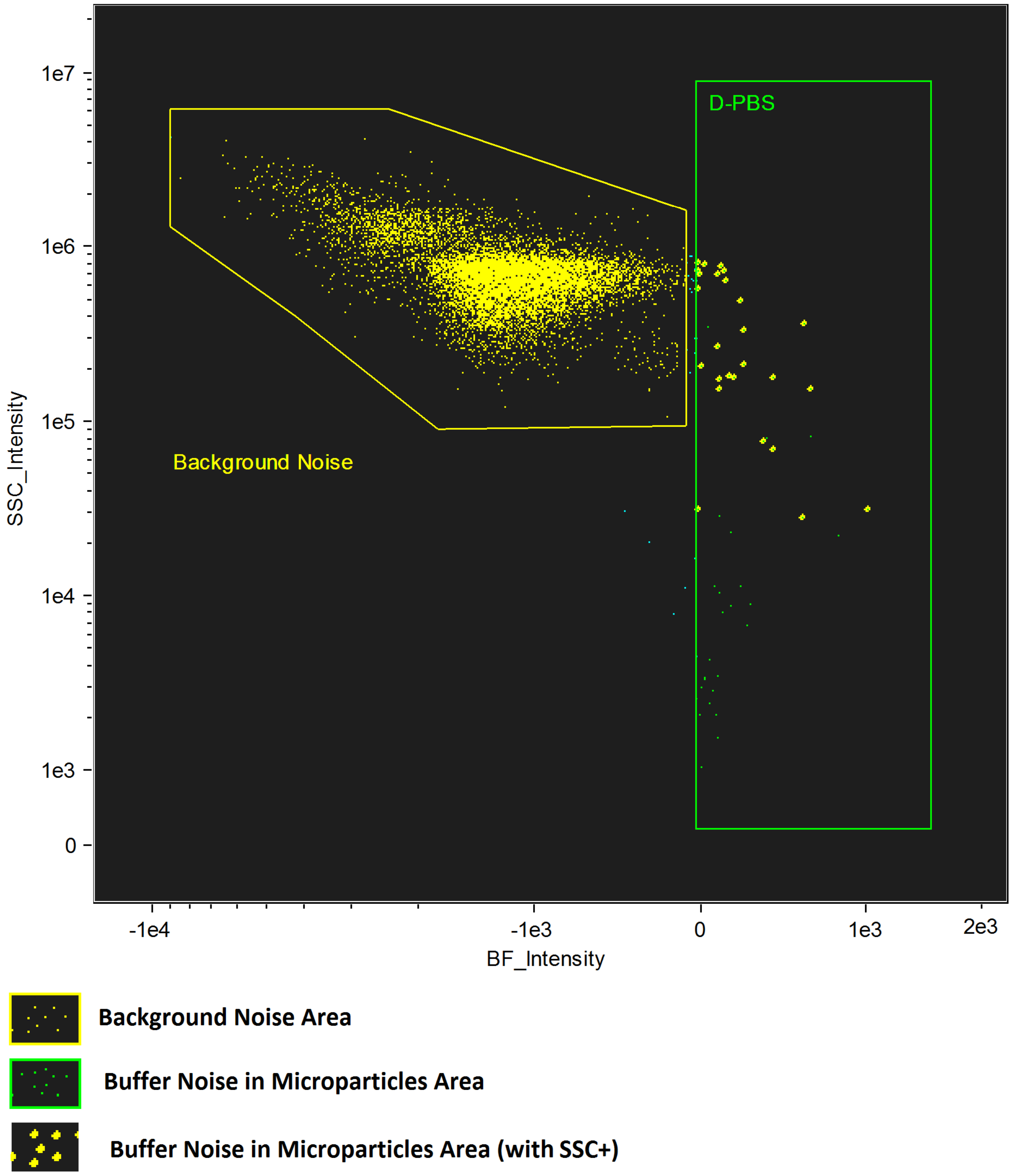

Fig. S3 Background analysis by ImageStreamX Mk II cytometery. The electronic noise has been reduced by the background determination (Background Noise). To reduce the interference of particle debris or other contaminates D-PBS used for analyses was filtered with common 0.2 µm milipore filter
